# A Risk Signature with Autophagy-Related Long Noncoding RNAs for Predicting the Prognosis of Clear Cell Renal Cell Carcinoma: Based on the TCGA Database and Bioinformatics

**DOI:** 10.1155/2021/8849977

**Published:** 2021-05-07

**Authors:** Yundong Xuan, Weihao Chen, Kan Liu, Yu Gao, Shidong Zuo, Baojun Wang, Xin Ma, Xu Zhang

**Affiliations:** ^1^Medical School of Chinese PLA, Beijing 100853, China; ^2^Department of Urology, The Third Medical Centre, Chinese PLA (People's Liberation Army) General Hospital, Beijing 100853, China

## Abstract

**Background:**

Disorders of autophagic processes have been reported to affect the survival outcome of clear cell renal cell carcinoma (ccRCC) patients. The purpose of our study was to identify and validate the candidate prognostic long noncoding RNA signature of autophagy.

**Methods:**

Transcriptome profiles were obtained from The Cancer Genome Atlas. The autophagy gene list was obtained from the Human Autophagy Database. Based on coexpression analysis, we obtained a list of autophagy-related lncRNAs (ARlncRNAs). GO enrichment analysis and KEGG pathway analysis were conducted to explore the functional annotation of these ARlncRNAs. Univariate and multivariate Cox regression analyses were conducted to elucidate the correlation between overall survival and the expression level of each ARlncRNAs. We then established a prognostic signature that was a linear combination of the regression coefficients from the multivariate Cox regression model (*β*) multiplied by the expression levels of the respective ARlncRNAs in the training cohort. The predictive performance was tested in the validation cohort. Additionally, the independence of the risk signature was assessed, and the relationship between the risk signature and conventional clinicopathological features was explored.

**Results:**

Seven autophagy-related lncRNAs with prognostic value (SNHG3, SNHG17, MELTF-AS1, HOTAIRM1, EPB41L4A-DT, AP003352.1, and AC145423.2) were identified and integrated into a risk signature, dividing patients into low-risk and high-risk groups. The risk signature was independent of conventional clinical characteristics as a prognostic indicator of ccRCC (HR, 1.074, 95% confidence interval: 1.036-1.113, *p* < 0.001) and was valuable in the prediction of ccRCC progression.

**Conclusion:**

Our risk signature has potential prognostic value in ccRCC, and these ARlncRNAs may play a significant role in ccRCC tumor biology.

## 1. Introduction

Renal cell carcinoma (RCC), a principal malignancy of the renal tubular epithelium, ranks third among urinary cancers1 [1]. Characterized by multifarious genetic features [2], RCC is composed of different histopathologic subtypes, of which clear cell renal cell carcinoma (ccRCC) represents the principle pathologic subtype, accounting for 70% to 80% of RCCs [3]. The prognosis of ccRCC varies between patients with complicated genetic mutations, such as von Hippel-Lindau (VHL), PBRM1, and BAP1 [4]. Surgery remains the principal treatment for ccRCC due to its therapeutic value, but 40% of ccRCC patients will eventually suffer distant metastasis [5], and few exhibit a positive response to radiotherapy or chemotherapy [6]. Neither immunologic nor targeted therapy can definitely benefit ccRCC patients [7]. Therefore, predicting the progression and prognosis of ccRCC by seeking novel effective biomarkers might facilitate the therapeutic schedule and the evaluation of survival status.

Autophagy, a process in which cells engulf proteins and organelles by forming double-membraned autophagic vesicles where degradation occurs, is regarded as a recycling of organelles and an adaptation of metabolism. Autophagy plays a tumor-suppressor role by inhibiting the selection and expansion of tumor-initiating cells early in tumor development [8]. However, increasing evidence in established tumors suggests that autophagy can help cope with environmental or intracellular stresses, such as hypoxia, nutrient shortage, or cancer therapy, thereby promoting tumor growth [9–11]. Although an increasing number of studies have sought to identify novel potential targets by probing the autophagy pathway, and autophagic drugs have been reported to induce renal cancer cell death, the mechanism is still to be clarified [12].

Long noncoding RNAs (lncRNAs), a variety of noncoding RNA, participate in many cellular processes with multitudinous functions by modulating gene expression at the epigenetic, transcriptional, and posttranscriptional levels [13–15]. Accumulating evidence suggests that lncRNAs can target autophagy-related genes at both the transcriptional and posttranscriptional levels to regulate the autophagy pathway, and lncRNAs regulate various proteins that function in the autophagy process [16, 17]. Dysregulated lncRNAs are involved in ccRCC progression and dissemination [18]. It has been suggested that autophagy-related lncRNAs can exert their action in tumor regulation [19].

The potential value of autophagy-related lncRNAs in evaluating the prognosis of ccRCC patients and their role as potential therapeutic targets have yet to be fully explored. Here, we sought to identify an autophagy-related lncRNA signature in ccRCC and to advance more personalized treatment guidelines for ccRCC through bioinformatic analysis.

## 2. Materials and Methods

### 2.1. Data Acquisition Processing

In our study, RNA-seq transcriptome data of which the workflow type was HTSeq-FPKM and clinical information data were downloaded from the TCGA database (https://portal.gdc.cancer.gov/) [20]. The lncRNA matrix and mRNA matrix were extracted from transcriptome profiling, respectively, by gene annotation. A list consisting of 232 autophagy genes was obtained from the Human Autophagy Database (HADb, http://autophagy.lu/clustering/index.html) (Table S1). Then, the expression matrix of 210 autophagy genes was extracted combing the mRNA matrix with autophagy genes list (Table S2), and 21 genes were abandoned due to lacking expressing information. In addition, the clinicopathological features included survival status, survival time, age, sex, International Society of Urological Pathology (ISUP) grade, and American Joint Committee on Cancer (AJCC) stage.

### 2.2. Autophagy-Related lncRNAs Screening

The Pearson correlation test was performed to screen autophagy-related lncRNAs based on 210 autophagy genes and all lncRNAs as mentioned. A lncRNA with a correlation coefficient ∣*R* | >0.3 and *p* < 0.001 was considered an autophagy-related lncRNA. The “limma” package in R software was then used to screen [21] differentially expressed autophagy-related lncRNAs (ARlncRNAs) (Table S3).

### 2.3. Construction of a Coexpression Network of the Autophagy-Related lncRNAs and Building a Sankey Diagram

Firstly, the clinicopathological data and ARlncRNAs were merged. Then, we constructed a mRNA-lncRNA interaction network of prognostic ARlncRNAs by applying Cytoscape software 3.8.0 [22]. A Sankey diagram was built to describe the relationship between the autophagy genes and lncRNAs by using the R software packages “ggalluvial” [23] and “ggplot2” [24].

### 2.4. Enrichment Analysis via DAVID Bioinformatics Resources

In this study, we conducted enrichment analyses via DAVID web server, a free server resource (https://david.ncifcrf.gov/summary.jsp) to elucidate the biological functions of those lncRNAs. 17 differentially expressed genes and 19 ARlncRNAs (Table S4) were taken to perform the analyses. Gene Ontology annotation and KEGG pathway were both involved.

### 2.5. Establishment of the ARlncRNA Signature and Performance Evaluation

First, the entire cohort obtained from the TCGA dataset was randomly divided into two cohorts: a training cohort (266 patients) and a validation cohort (264 patients). The training cohort was used to establish the Cox regression risk signature, and the validation cohort was then used to assess the performance of the signature. Then, univariate Cox regression and Kaplan-Meier (KM) analysis were used to identify ARlncRNAs with prognostic value. Seven lncRNAs considered significant with a *p* value < 0.0001 by both analyses were included in the resulting model. Next, multivariate stepwise regression Cox analysis was performed to establish a prognostic risk model. The risk score was calculated as the sum of multivariate Cox regression coefficient (*β*)-weighted expression levels of lncRNAs: Risk score = *β*gene(1) × expression level of gene(1) + *β*gene(2) × expression level of gene(2) + ⋯+*β*gene(*n*) × expression level of gene(*n*). The training cohort was then separated into high-risk and low-risk groups based on the median risk score. We generated a receiver operating characteristic (ROC) curve to evaluate the predictive power of the risk score. The same analysis was adopted in the testing cohort to assess the performance of the signature. Additionally, we built a nomogram using the R package “rms” based on the results of multivariate Cox regression analysis to evaluate the prognosis of ccRCC. Lastly, the C-index and calibration curve were used to assess the performance value.

### 2.6. Independence Verification of the Risk Signature and Performance Evaluation

Univariate and multivariate Cox regression analyses were performed to investigate the independence of the risk signature as a predictive factor from the traditional clinical features (including age, sex, ISUP grade, and AJCC stage) in both the training and validation cohorts.

### 2.7. Statistical Analysis

In this study, all analyses were performed using R 4.0.2. A *p* value < 0.05 was considered statistically significant. The correlation matrix was constructed by R software based on Pearson correlation coefficients. The relationship between autophagy-related lncRNAs and overall survival was analyzed through the Kaplan-Meier curve, which was evaluated by the log-rank test. Time-dependent ROC curves were used to analyze the sensitivity and specificity of the prognostic prediction model. The nomogram was constructed with the regression coefficients based on the Cox analysis, and its performance was assessed by the c-index and calibration curve. Univariate and multivariate Cox regression analyses were performed to investigate the independence of the risk signature as a predictive factor, and Student's *t*-test was used to compare the clinicopathological features between different risk-score groups.

## 3. Results

### 3.1. Data Processing and Prognostic ARlncR Acquisition

**Figure 1:**
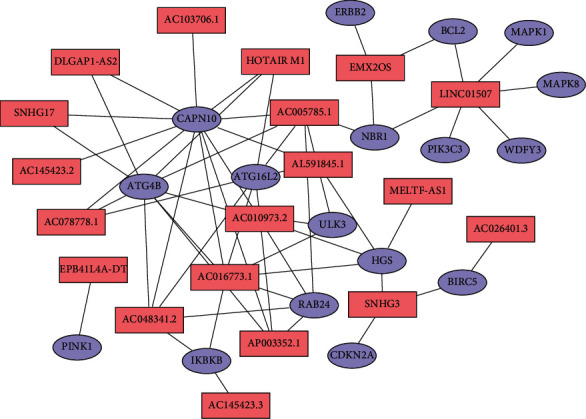
Network of prognostic lncRNAs with coexpressed autophagy genes in ccRCC. In the centric position, red nodes indicate lncRNAs, and blue nodes indicate autophagy genes. The coexpression network was visualized by CYTOSCAPE software 3.8.0.

**Figure 2:**
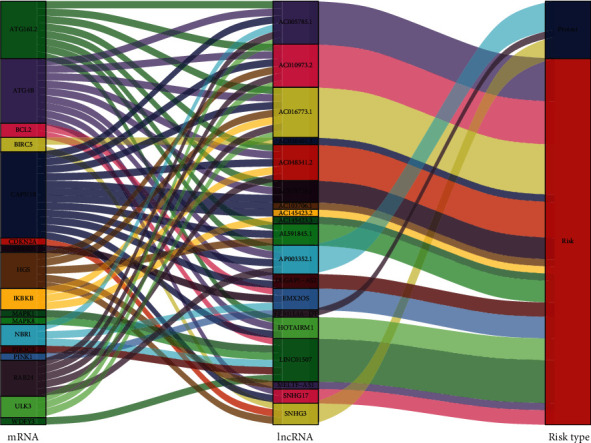
Correlation between ARlncRNAs and autophagy-related genes. Left bar: mRNA; middle bar: lncRNA; right bar: risk type. mRNA: messenger RNA; lncRNA: long noncoding RNA.

We obtained transcriptome data and clinical data from the TCGA dataset. The basic clinical characteristics of the ccRCC patients in the TCGA database are shown in Table 1. Based on a total of 232 autophagy-related genes from the Human Autophagy Database (HADb, http://autophagy.lu/clustering/index.html), we screened 813 autophagy-related lncRNAs. Nineteen ARlncRNAs closely connected to prognosis were identified in the training cohort through univariate Cox regression analysis (*p* value < 0.01), and then, a network of prognostic 19 lncRNAs with coexpressed 17 autophagy genes in ccRCC and a Sankey diagram for visualization were built. The results are shown in Figures 1 and 2.

### 3.2. Enrichment Analyses

**Figure 3:**
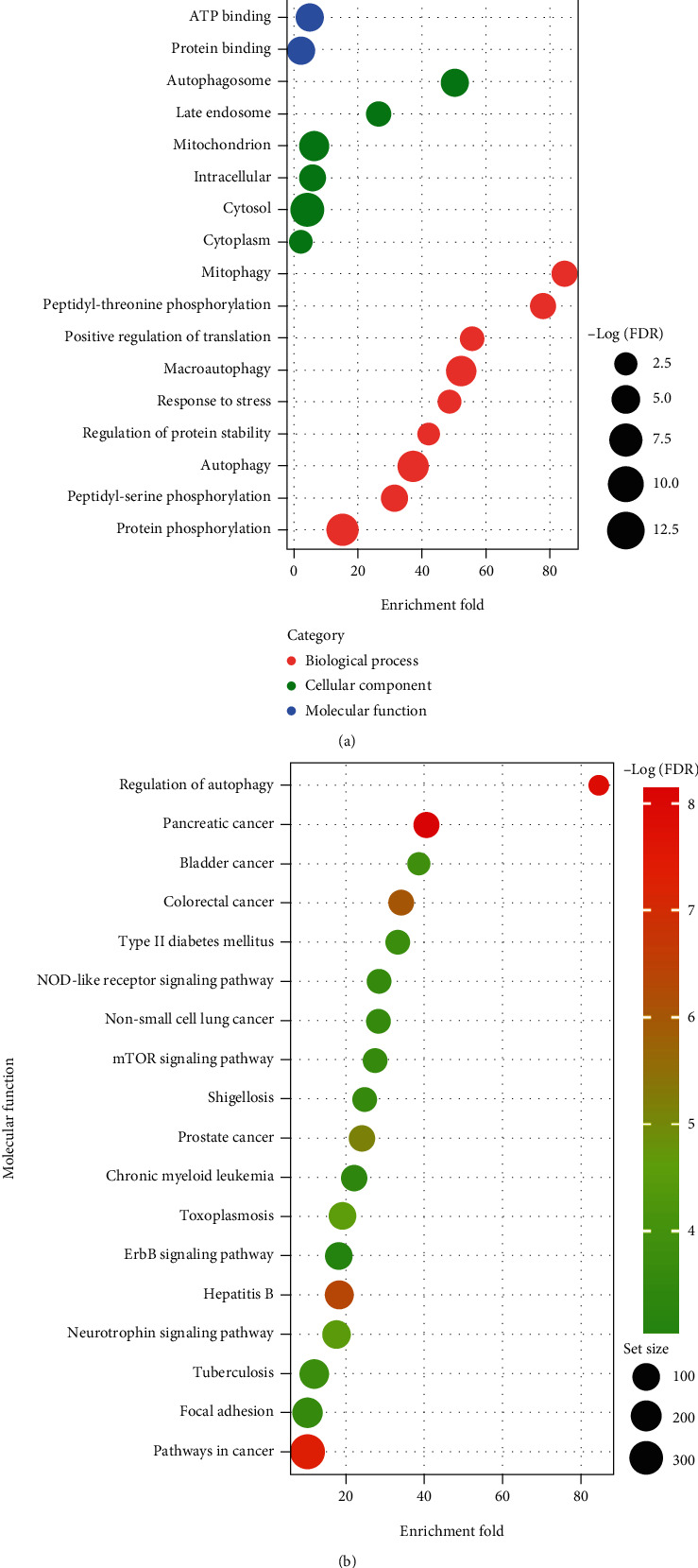
GO and KEGG pathway enrichment analysis. (a) GO enrichment analysis. Red nodes represent the changes in biological processes (BPs), green nodes represent the changes in cell components (CCs), blue nodes represent the changes in molecular functions (MFs). The *x*-axis represents enrichment fold. (b) KEGG pathway enrichment analysis. The node color changes gradually from red to green in ascending order according to the –log(FDR) va. The size of each node represents the number of gene sets.

To further identify the Gene Ontology (GO) annotation and KEGG pathway in which the above lncRNAs were enriched, GO term and KEGG pathway enrichment analyses were performed via DAVID functional annotation tool. The visualization of results was achieved by “ggplot2” packages in R software. GO analysis showed that changes in the biological processes (BPs) of autophagy-related lncRNAs were enriched in mitophagy, peptidyl-threonine phosphorylation, positive regulation of translation, macroautophagy, and others (Table 2, Figure 3(a)). Changes in cell components (CCs) included the autophagosome, late endosome, and mitochondrion (Table 2, Figure 3(a)). Changes in molecular functions (MFs) were mainly enriched in kinase activity, protein kinase activity, protein serine or threonine activity, and others (Table 2, Figure 3(a)). KEGG pathway enrichment analysis indicated that those autophagy-related lncRNAs were involved in multiple tumor progressions and signaling pathways such as NOD-like receptor signaling pathway and mTOR signaling pathway (Table S5, Figure 3(b)).

### 3.3. Establishment of the Autophagy-Related lncRNA Signature for ccRCC

**Figure 4:**
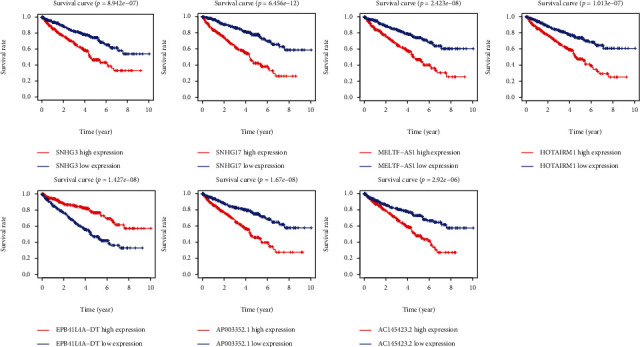
Kaplan-Meier survival curves for the 7 prognostic lncRNAs for ccRCC. The 7 autophagy-related lncRNAs were found to be of value in predicting prognosis in ccRCC patients.

**Figure 5:**
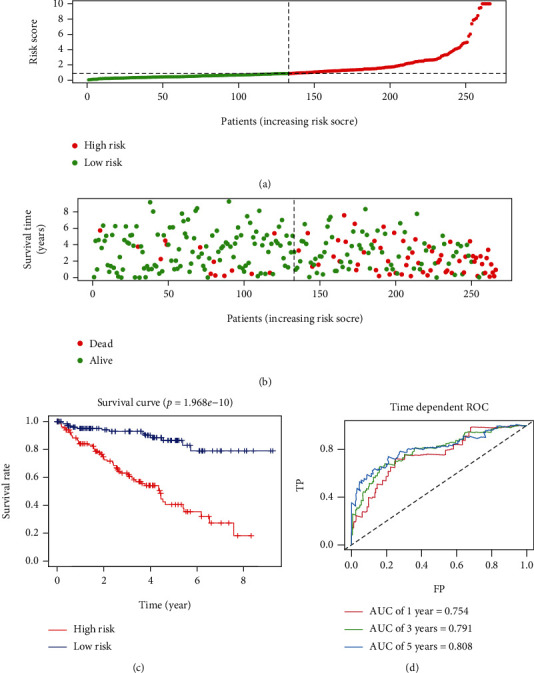
Prognostic analyses between the patients in the high-risk group and low-risk group in the training cohort. (a) Risk score distribution of patients from the prognostic signature. (b) Survival status scatter plots for patients in the prognostic signature (green dots: alive; red dots: death). (c) The Kaplan-Meier plot (high-risk vs. low-risk group) of the training cohort. (d) Time-dependent receiver operating characteristic curves assessed the predictive efficiency of the risk score for the training cohort. AUC: area under the curve; FP: false positive; TP: true positive.

To improve our prognostic ability further, we employed stepwise multivariate Cox regression analysis to further evaluate the aforementioned ARlncRNAs. As a result, 7 lncRNAs were eventually pulled out to construct the signature. Herein, the risk score was assigned using a linear combination of the expression levels of the 7 identified lncRNAs weighted by their regression coefficients (*β*) (Table 3, Figure 4). Then, the ccRCC patients were divided into high-risk and low-risk groups around the median risk score. As a result, the risk score distribution of the patients on the basis of the prognostic signature is shown in Figure 5(a). Survival status scatter plots for the patients based on the prognostic model are shown in Figure 5(b), indicating that patients in the high-risk group had a higher mortality than those in the low-risk group. A significant difference in overall survival between the two groups was seen (Figure 5(c), *p* < 0.0001), and the AUCs at one, three, and five years were 0.754, 0.791, and 0.808, respectively (Figures 5(d)).

### 3.4. Evaluation of the Prediction Performance of the Signature

**Figure 6:**
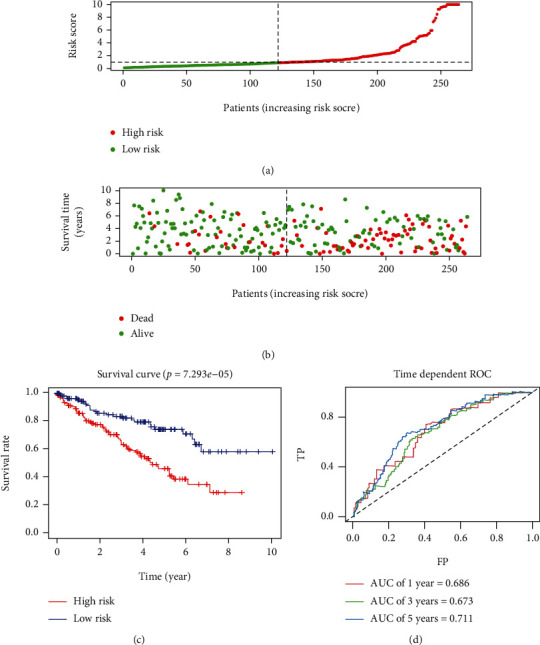
Prognostic analyses between the patients in the high-risk group and low-risk group in the validation cohort. (a) Risk score distribution of patients from the prognostic signature. (b) Survival status scatter plots for patients in the prognostic signature (green dots: alive; red dots: death). (c) The Kaplan-Meier plot (high-risk vs. low-risk group) of the training cohort. (d) Time-dependent receiver operating characteristic curves to assess the predictive efficiency of the risk score in the training cohort. AUC: area under the curve; FP: false positive; TP: true positive.

**Figure 7:**
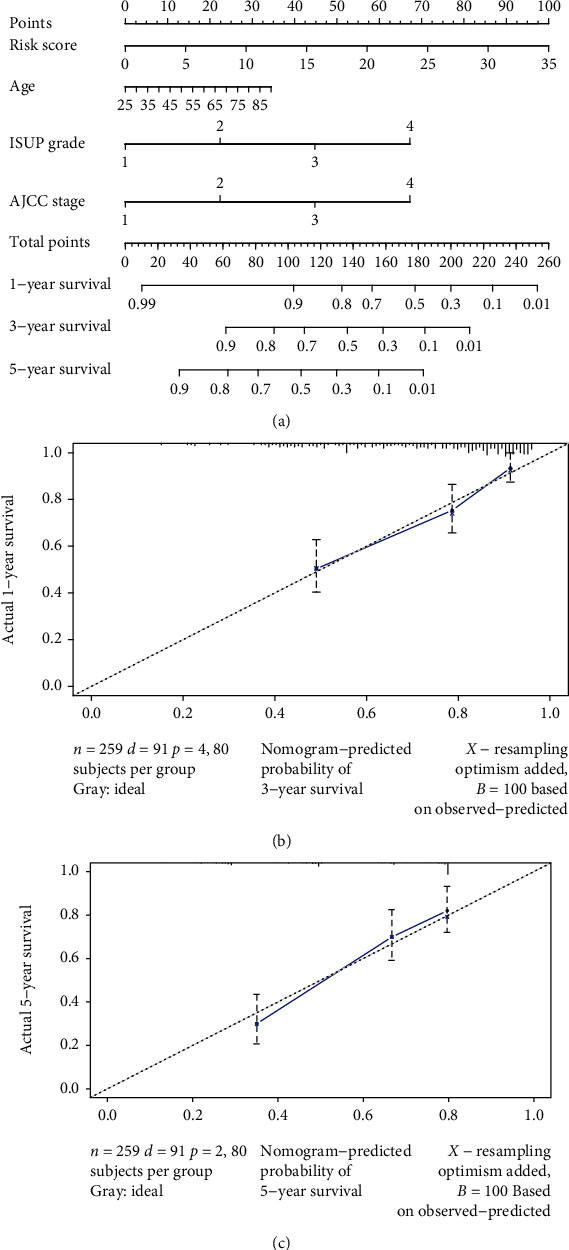
(a) Nomogram predicting 1-, 3-, and 5-year overall survival for patients with ccRCC. The calibration curve for predicting patient survival at (b) 3 years and (c) 5 years in the TCGA datasets. The nomogram-predicted probability of overall survival is plotted on the *x*-axis; actual overall survival is plotted on the *y*-axis.

Next, we assessed the predictive ability of the prognostic signature in the validation cohort to further evaluate its performance. The risk scores of the above 7 lncRNAs were recalculated for each patient in the validation cohort. As shown in (Figure 6(a)), the risk score distribution of the patients based on the prognostic model presented similar results as in the training cohort. Survival status scatter plots for the patients on the basis prognostic model are presented in (Figure 6(b)). Survival differences were significant in the validation cohort (Figure 6(c)), *p* < 0.0001). The AUCs at one, three, and five years were 0.686, 0.673, and 0.711, respectively (Figures 6(d)). In addition, we constructed a nomogram to forecast the overall survival in the entire cohort with ccRCC according to the clinicopathological features and risk scores (Figure 7(a)), of which the C-index was 0.773. The calibration curve for the nomogram suggested good performance. Thus, the nomogram proved to be of value in the prediction of the prognosis of ccRCC patients (Figures 7(b) and 7(c)).

### 3.5. Independence Verification of the Signature as a Prognostic Predictor

**Figure 8:**
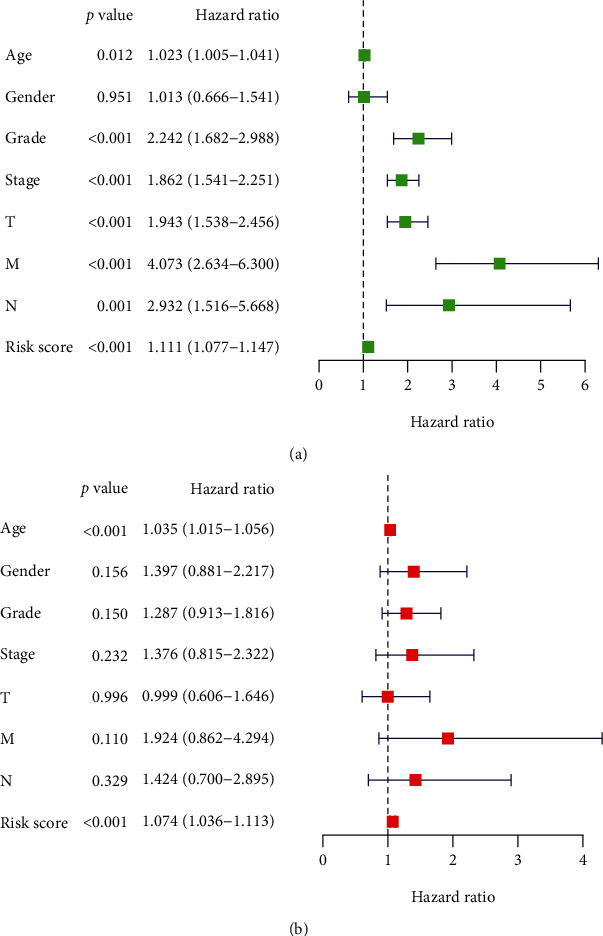
Univariate (a) and multivariate (b) Cox regression analyses for the TCGA cohort of ccRCC patients.

To further investigate whether the risk score could be predictive for ccRCC independent of conventional clinicopathological features (age, sex, ISUP grade, and AJCC state), univariate and multivariate Cox regression analyses were run. The risk score proved to be independent of the aforementioned clinical features in the entire cohort (Figure 8) (HR, 1.074, 95% confidence interval: 1.036-1.113, *p* < 0.001). The independence of the signature was further validated by stratified clinical features.

**Figure 9:**
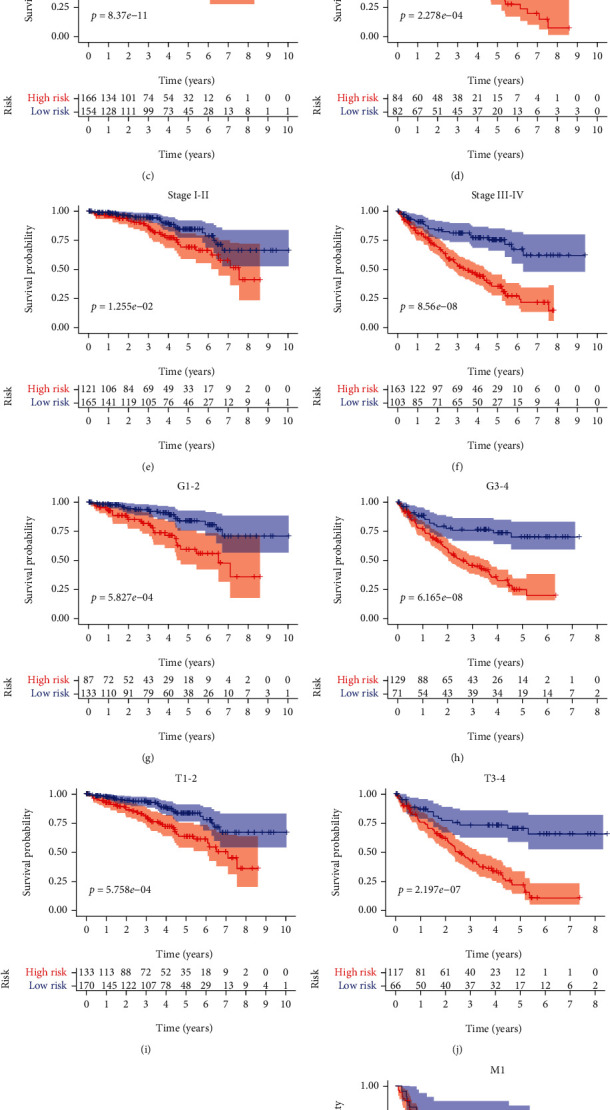
Kaplan-Meier survival curves for the high-risk group and low-risk group stratified by clinicopathological features. (a, b) Sex. (c, d) Age. (e, f) Stage. (g, h) Grade. (i, j) T stage. (k, l) M stage. T: tumor size; M: metastasis.

We conducted analyses to explore the value of the ARlncRNA signature in different clinicopathological subgroups, including age, sex, ISUP grade, AJCC stage, T stage, and M stage. Comparing the two results of each stratified feature revealed that the overall survival time of the high-risk group was shorter than that of the low-risk group in all clinicopathological subgroups (Figure 9). These results further indicated that the autophagy-related lncRNA signature can independently predict the prognosis of ccRCC patients.

### 3.6. Exploring the Predictive Value of the Signature for Tumor Progression

**Figure 10:**
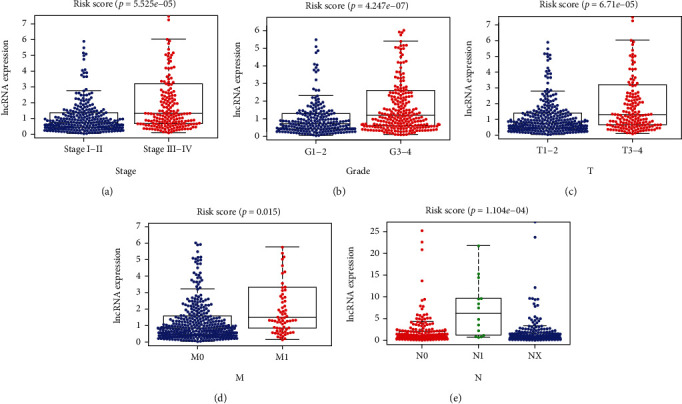
Evaluation of the predictive value of the signature for progression. (a) Stage. (b) Grade. (c) T stage. (d) M Stage. (e) N stage. T: tumor size; M: metastasis; N: lymph node metastasis.

In order to get a deeper insight into the predictive value of the signature for ccRCC patients with regard to tumor progression, correlation analyses between the autophagy-related prognostic signature and clinicopathological features were performed. The risk score of stage III–IV ccRCC was higher than that of stage I–II (*p* = 5.525*e* − 05, Figure 10(a)). The risk score of G3–4 was higher than that of G1–2 (p = 4.247e−07, Figure 10(b)). The risk score of T3–4 was higher than that of T1–2 (*p* = 6.71*e* − 05, Figure 10(c)). The risk score of M1 was higher than that of M0 (*p* = 0.015, Figure 10(d)), and the risk score of N1 was higher than that of N0 (*p* = 1.104*e* − 04, Figure 10(e)). Taken together, these results indicated that as the risk score increased, the malignancy of ccRCC increased. Thus, the prognostic signature was of unique value for predicting the progression of ccRCC.

### 3.7. Relationships between the Prognostic ARlncRNAs and Clinicopathological Features

Subsequently, we investigated the connection between those ARlncRNAs and clinicopathological features. The purpose was to help develop a deeper understanding of the autophagy process, and the results shown in Table 4 revealed that each of those prognostic ARlncRNAs was significantly associated with clinicopathological features that are closely connected with tumor progression, including ISUP grade, AJCC stage, T stage, and N stage. SNHG17 and AP003352.1 were significantly associated with age. However, only EPB41L4A-DT was significantly associated with sex. Overall, these results indicated that the aforementioned ARlncRNAs can promote tumor progression.

## 4. Discussion

ccRCC is well known for its heterogeneity, exhibiting molecular diversity, morphological variability, and metabolic reprogramming. Late diagnosis without early warning signs and limited response to chemotherapy or radiotherapy are the main culprits of poor prognosis [25]. Although novel biomarkers, especially autophagy-related genes and molecules, are emerging as predictive factors thanks to in-depth cancer genetics and molecular biology discoveries [26, 27], the value of autophagy-related lncRNAs as prognostic indicators has not been addressed. Unlike previous studies that focused on the role of autophagy-related genes in tumorigenesis and progression [28–30], our study is aimed at improving prognostic prediction by finding autophagy-related lncRNAs associated with the poor prognosis of ccRCC through comprehensive bioinformatics analysis in TCGA databases.

We first identified 813 lncRNAs on the basis of the lncRNA-autophagy gene coexpression network. By using a univariate Cox regression model, we identified 19 ARlncRNAs associated with the prognosis of ccRCC patients. Seven ARlncRNAs were further screened using multivariate Cox regression analysis, including SNHG3, SNHG17, MELTF-AS1, HOTAIRM1, EPB41L4A-DT, AP003352.1, and AC145423.2. GO analysis was conducted to discover the main biological characteristics of these ARlncRNAs. Next, we constructed a risk score-based prognostic signature that separated ccRCC patients into low-risk and high-risk groups. The OS time in the high-risk group was shorter than that in the low-risk group. The prediction performance was validated in the validation cohorts.

Furthermore, through univariate and multivariate Cox regression analyses, the risk score based on the signature was shown to be a prognostic factor for ccRCC independent of conventional clinicopathological features (age, sex, ISUP grade, and AJCC stage). Further evaluation demonstrated that the ARlncRNA signature can independently predict the progression of ccRCC, which means that the higher the risk score was, the worse the prognosis and the greater the degree of malignancy were. Finally, we established a nomogram based on the risk score of the signature, and the C index and calibration curve indicated that the predictive performance of the nomogram was good. Overall, these results indicate that our ARlncRNA signature can play an important role in predicting the prognosis of ccRCC patients.

Autophagy is significantly connected to the prognosis of cancer; however, the complicated process and the numerous molecular interactions make autophagy play contradictory roles in cancer [31]. Long noncoding RNA small nucleolar RNA host gene 17 (SNHG17) was reported to be a critical regulator of tumorigenesis, and studies have reported its role in promoting tumor invasion and proliferation by activating the PI3K/AKT pathway [32, 33]. Jiang et al. [34] have reported that MELTF-AS1 can serve as a prognostic indicator and is associated with immunological processes. Our study further validates its prognostic value in association with the autophagy process, indicating that MELTF-AS can play distinct roles in multiple physio-pathological processes. HOTAIRM1 can promote glioblastoma progression [35], but in ccRCC, it is downregulated, serving as a suppressor of HIF1-dependent angiogenic pathways [36]. Thus, in vivo and in vitro experiments are required to illustrate the full effects of HOTAIRM1. Although we are the first to reveal the predictive value of EPB41L4A-DT, AP003352.1, and AC145423.2, their actual functions remain to be determined.

The common methods to detect lncRNA for clinical applications currently include microarray, lncRNA sequencing, and quantitative RT-PCR. Although the microarray can facilitate large-scale detections, its cost is relatively high [37]. LncRNA sequencing enables the detection to be more efficient and break the limit of traditional methods but leaving the library construction work tedious and expensive [38]. Quantitative RT-PCR is also a common method used to detect lncRNAs. It has been widely used because of its simplicity and low cost though it is still a low-throughput and low-specificity method [39]. Given those shortages, a comprehensive and personalized detection method of lncRNAs with further research achievements could be actually integrated into future precision medicine strategies.

Despite our novel findings, there are still limitations to this study. First, more basic experiments are required to clarify the mechanisms of action of ARlncRNAs in ccRCC tumor progression. Second, an external data set should be employed for validation, instead of our limited internal data set, to assess the consistency, reliability, and applicability of the autophagy-related signature.

## 5. Conclusion

In conclusion, we established an autophagy-related signature that independently predicted the prognosis of ccRCC patients. It can be used to guide individualized treatment regimens. Our study could help broaden the understanding of autophagy-related lncRNAs and narrow the gap between theoretical research and clinical practice, but the underlying mechanisms still urgently need to be understood to clarify the importance of our findings.

## Figures and Tables

**Table 1:** Clinical characteristics of ccRCC patients in the TCGA database.

Characteristics		Total	%
Age at diagnosis (y)		58 (26~90)	
Gender	Female	191	35.57
	Male	346	64.43
Stage	I	269	50.37
	II	57	10.67
	III	125	23.41
	IV	83	15.55
Grade	G1	14	2.65
	G2	230	43.48
	G3	207	39.13
	G4	78	14.74
T stage	T1	275	51.21
	T2	69	12.85
	T3	182	33.89
	T4	11	2.05
N stage	N0	240	93.39
	N1	17	6.61
M stage	M0	426	84.36
	M1	79	15.64

**Table 2:** Functional enrichment analyses of the prognostic autophagy-related lncRNAs.

Category	ID	Description	*p* value	FDR	Genes
Biological process	GO:0000422	Mitophagy	4.97*E*-04	0.038125	PINK1, CDKN2A, ATG4B
Biological process	GO:0018017	Peptidyl-threonine phosphorylation	5.87*E*-04	0.038125	MAPK8, BCL2, MAPK1
Biological process	GO:0045727	Positive regulation of translation	1.14*E*-03	0.063524	PINK1, ERBB2, MAPK1
Biological process	GO:0016236	Macroautophagy	4.78*E*-05	0.006217	PINK1, NBR1, ATG4B, PIK3C3
Biological process	GO:0006950	Response to stress	1.50*E*-03	0.073488	PINK1, MAPK8, MAPK1
Biological process	GO:0031647	Regulation of protein stability	1.98*E*-03	0.002612	CDKN2A, BCL2, MAPK1
Biological process	GO:0006914	Autophagy	6.36*E*-06	0.001240	PINK1, NBR1, ATG4B, PIK3C3
Biological process	GO:0018105	Peptidyl-serine phosphorylation	2.10*E*-04	0.020483	PINK1, MAPK8, BCL2, MAPK1
Biological process	GO:0006468	Protein phosphorylation	2.47*E*-06	9.62E-04	IKBKB, PINK1, MAPK8, ERBB2, MAPK1, BICR5, PIK3C3
Cellular component	GO:0005776	Autophagosome	1.4*E*-03	0.029687	NBR1, RAB24, WDFY3
Cellular component	GO:0005770	Late endosome	5.0*E*-03	0.069394	NBR1, MAPK1, PIK3C3
Cellular component	GO:0005739	Mitochondrion	6.94*E*-05	0.002878	PINK1, MAPK8, CDKN2A, CAPN10, RAB24, BCL2, MAPK1
Cellular component	GO:0005622	Intracellular	6.37*E*-04	0.017625	IKBKB, MAPK8, HGS, CAPN10, RAB24, MAPK1, PIK3C3
Cellular component	GO:0005737	Cytoplasm	5.0*E*-03	0.069395	IKBKB, PINK1, CDKN2A, HGS, ERBB2, BCL2, ULK3, ATG4B, MAPK1, BIRC5, WDFY3
Molecular function	GO:0016301	Kinase activity	3.58*E*-08	2.68E-06	PINK1, MAPK8, CDKN2A, HGS, ERBB2, MAPK1, PIK3C3,
Molecular function	GO:0004672	Protein kinase activity	2.28*E*-04	0.006804	IKBKB, PINK1, ERBB2, ULK3, PIK3C3
Molecular function	GO:0004674	Protein serine/threonine kinase activity	0.00406432.72*E*-04	0.020892 0.006803	BIRC5, PINK1 IKBKB, PINK1, MAPK8, ULK3, MAPK1
Molecular function	GO:0046982	Protein heterodimerization activity	0.007382	0.092273	IKBKB, ERBB2, BCL2, BIRC5
Molecular function	GO:0005524	ATP binding	1.2*E*-03	0.017773	IKBKB, PINK1, MAPK8, ERBB2, ULK3, MAPK1, PIK3C3
Molecular function	GO:0004861	Protein binding	8.21*E*-04	0.015391	CDKN2A, IKBKB, PINK1, MAPK8, HGS, NBR1, ERBB2, RAB24, ULK3, BCL2, BIRC5, ATG4B, MAPK1, PIK3C3, WDFY3

**Table 3:** Multivariate Cox regression analysis of prognostic autophagy-related genes.

ID	Coef	HR	HR.95 L	HR.95 H	*p* value
SNHG3	-0.647762	0.523215	0.281957	0.970904	0.040017
SNHG17	0.966878	2.629724	1.453059	4.759232	0.001400
MELTF-AS1	0.528276	1.696006	1.094015	2.629247	0.018193
HOTAIRM1	0.496232	1.642520	1.098048	2.456972	0.015726
EPB41L4A-DT	-1.617247	0.198444	0.104238	0.377789	8.51E-07
AP003352.1	-0.910912	0.402157	0.168855	0.957802	0.039653
AC145423.2	0.511553	1.667880	0.923432	3.012484	0.089907

**Table 4:** Relationships between the prognostic ARGs and clinicopathological features.

lncRNA		Age		Sex		Grade		Stage		T stage		M stage	
		≤65	>65	Female	Male	G1-2	G3-4	I-II	III-IV	T1-2	T3-4	M0	M1
*N*				166	323	221	268	289	200	306	183	412	77
SNHG3	*t* value	1.81		0.1246		3.660		4.565		4.405		2.791	
	*p* value	0.071		0.909		0.0003		<0.0001		<0.0001		0.0054	
SNHG17	*t* value	2.24		1.17		3.152		5.149		4.959		3.228	
	*p* value	0.0256		0.0017		0.0017		<0.0001		<0.0001		0.0013	
MELTF-AS1	*t* value	1.427		1.135		4.319		6.689		6.185		4.611	
	*p* value	0.1542		0.2571		<0.0001		<0.0001		<0.0001		<0.0001	
HOTAIRM1	*t* value	1.082		0.9008		3.480		4.407		4.353		2.503	
	*p* value	0.28		0.3682		0.0005		<0.0001		<0.0001		0.0126	
EPB41L4A-DT	*t* value	0.6985		2.701		5.920		5.826		7.712		4.475	
	*p* value	0.4852		0.0072		<0.0001		<0.0001		<0.0001		<0.0001	
AP003352.1	*t* value	2.207		0.6670		2.649		4.72		4.424		2.814	
	*p* value	0.028		0.5051		0.0083		<0.0001		<0.0001		0.0051	
AC145423.2	*t* value	0.2175		0.2175		3.921		3.742		2.836		2.302	
	*p* value	0.8279		0.8279		0.0001		0.0002		0.0048		0.0218	

## Data Availability

The data included in the current study were available in TCGA database (https://cancergenome.nih.gov/), the Human Autophagy Database (http://autophagy.lu/clustering/index.html), and DAVID (https://david.ncifcrf.gov/summary.jsp).

## Supplementary Materials

Supplementary Materials Table S1: 232 autophagy genes obtained from the Human Autophagy Database. Table S2: 210 autophagy genes expression matrix. Table S3: 813 autophagy-related lncRNAs. Table S4: 17 autophagy genes and 19 lncRNAs used to enrichment analyses. Table S5: results of enrichment via DAVID web serve.
